# Characterization and Research on Mechanical Properties of Bamboo Plastic Composites

**DOI:** 10.3390/polym10080814

**Published:** 2018-07-25

**Authors:** Yu Xian, Dongjuan Ma, Cuicui Wang, Ge Wang, Leemiller Smith, Haitao Cheng

**Affiliations:** 1 The College of Forestry, Shanxi Agricultural University, Taigu 030801, China; xianyu_sxau@126.com; 2 Wood Science and Technology, International Centre for Bamboo and Rattan, Beijing 100102, China; wang_icbr@126.com; 3 State Grid Shanxi Electric Power Research Institute, Taiyuan 030001, China; mdj_sgcc@126.com; 4 Ministry of Education Key Laboratory of Wooden Material Science and Application, Beijing Forestry University, Beijing 100083, China; wang_bjfu@126.com; 5 Mechanical Energy Engineering Department, University of North Texas, Denton, TX 76207-7102, USA; leemiller.smith27@gmail.com

**Keywords:** bamboo plastic composites, mechanical properties, essential work of fracture methodology

## Abstract

The focus of this study was to observe the mechanical properties of bamboo plastic composites (BPCs) with bamboo pulp fiber (BPF) or white mud (WM). The essential work of fracture (EWF) methodology was used to characterize the impact toughness of BPCs. The results revealed an increase in flexural, tensile and impact properties, when adding the BPF in the BPCs. While the flexural properties of WM-reinforced BPCs revealed increasing, there was a decrease in tensile and impact strength. In an impact strength analysis study, BPF-filled BPCs showed excellent impact property over WM-filled BPCs; scanning electron microscopy (SEM) helps to explain impact fracture behavior of BPCs. EWF analysis of impact results showed that the specific essential work of fracture (*w_e_*) increased significantly with the amount of BPF used in BPCs but decreased with the increase of WM in the BPCs. There was similar variation for the non-essential plastic work (*βw_p_*) of BPCs. This result indicates that the fracture initiation and fracture propagation of BPCs are different.

## 1. Introduction

With the national economy and further improvement of people’s living standards, the mass consumption demands of global resources are gradually increasing, which has triggered a series of environmental sustainability and material availability [1]. As well as the rise in public awareness of environmental protection, while the forest resources were limited, this led to the demand for various wood products to keep soaring. Thus, there is an urgent need to find alternative materials to substitute for shrinking timber resources in China [2]. Therefore, the research about wood plastic composites (WPCs) has drawn the attention of the public.

WPCs are a kind of biocomposites, which are formed by wood, wood flour or other lignocellulose fibers with various plastics in different combining paths. Compared with other wood materials, WPCs possess low maintenance costs and environment-friendly performance, which has seen its application across residential markets, construction, manufacturing, automotive and outdoor structures [3,4]. Low monolithic properties in WPCs has resulted in limited use in more demanding fields, due to poor interface compatibility between the wood flour and polymer matrix [5]. One way to enhance the interfacial interaction is with the addition of maleic anhydride grafted polyethylene (MAPE), which is a renowned and widely utilized graft polymer as a modifier to boost the compatibility between wood flour and plastic blends, meanwhile the morphology and construction of natural fibers have great influence on the properties of WPCs. The influence of fiber content and length, bamboo and glass fiber mixture ratio in polypropylene composite on tensile and flexural properties, fatigue fracture behavior under periodic loading has been reported. Because of the synergistic effects between the glass fiber and wood fiber in the composite, the tensile and flexural properties of composites revealed an outstanding improvement due to the aspect ratio of glass fiber plays on the 3-D network architecture of the composite [6,7,8]. The critical length of the fibers contributes greatly to the mechanical performances, which suggest that reasonable selection of wood fiber might be a useful tool for improving overall performance, other studies have shown that kenaf, jute, sisal and cellulose fibers are all prominent natural fibers used in industry as reinforcement materials [9,10]. He et al. studied the mechanical performance of WPCs prepared by blending tea residue with high-density polyethylene (HDPE) [11]. Ebadi et al. investigated the physical and mechanical properties of WPCs based on wood powder to beverage storage carton waste in varying proportions [12]. Kajaks et al. have studied the influence of modifiers on the rheological property and thermal stability property of plywood production residues polypropylene composites [13]. Some related works were done using WPCs based on cellulosic waste and mineral filler [14,15]. The significant increase in modulus of elasticity and strength of WPCs is recognized and is reasonably understood. Numerous published studies have been shown due to the great prospect of WPCs based on plant fibers [16,17,18]. However, few studies have been performed on impact toughness behavior of WPCs. Generally, the impact toughness behavior of WPCs was evaluated by empirical tests, such as notched and un-notched impact tests being used to analysis impact property [19]. However, the fracture energies of WPCs based on these tests, which are dependent on specimen geometry and test configuration etc., therefore did not reflect the true impact fracture behavior of composites [20]. The toughness and crack resistance behavior of composites can be given by fracture mechanics analysis. The fracture behavior of various composites has already been analyzed by the essential work of fracture (EWF) method [21]. Yilmaz et al. [22] investigated the fracture behavior of the acrylonitrile–butadiene-styrene/Polyamide 6 (ABS/PA6) blends reinforced short glass fiber and CaCO_3_ using EWF method, the results of which show that fracture behavior of the ABS/PA6 blends turned from ductile to brittle; this was due to ratio of reinforcement in the matrix increased. He et al. [20] studied fracture toughness of multilayered-structure materials based on the EWF method, which results showed that the number of layers have a great influence on the resistance to crack propagation of multilayered specimen.

Previous research has shown that bamboo pulp fiber (BPF) and white mud (WM) significantly improved the mechanical properties of polymer composites [23,24]. Bamboo residue fiber (BRF)-reinforced high-density polyethylene (HDPE) caused the increase of tensile and flexural properties, but BRF-reinforced HDPE had lower impact strength [25]. For this reason, BPF and WM are used to improve the mechanical properties of bamboo plastic composites (BPCs). Due to cost always being an important criterion that can restrict the development of a technology, this means that any new material or method developed takes this into account, which is a load of BRF and WM used can be used to reduce costs. Therefore, BPF and WM were used to reinforce BPCs that were manufactured using extrusion technology in this paper. The effect of BPF and WM loading on the flexural, tensile and impact properties were investigated with the macro test, the difference in the fracture toughness of BPCs was also studied by means of the EWF method based on the Charpy impact test results.

## 2. Essential Work of Fracture (EWF) Theory

**Figure 1:**
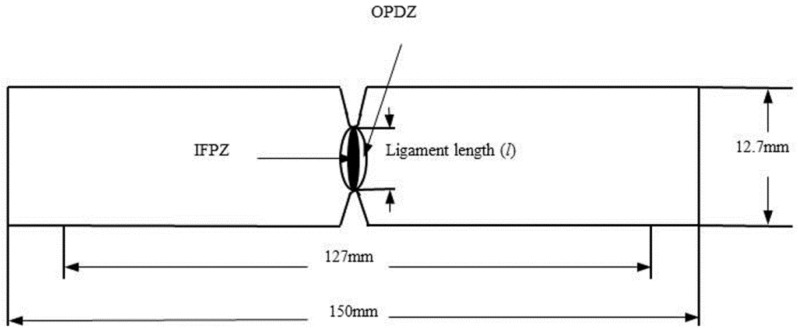
Schematic of double-edge notched impact specimen based on essential work of fracture (EWF). IFPZ and OPDZ denote inner fracture process zone and outer plastic deformation zone, respectively.

Double-edge notched impact specimens were prepared with different ligament lengths in this study, which are subjected to the impact experiments. Figure 1 shows the symmetry of eligibility of double-edge notched impact specimen, which will be used here.

The fracture behavior of various composites was analyzed based on the EWF method when it was proposed firstly [26]. The total work of fracture (*W_f_*) is divided into essential work of fracture (*W_e_*) and the non-essential (or plastic) work (*W_p_*), two components according to the energy partition, which is calculated from the relationship between force and displacement (*F*–*x*) curves. The total work of fracture (*W_f_*) can be calculated by Equation (1):

*W_f_* = *W_e_* + *W_p_* (1)

The first term is essential work of fracture. (*W_e_*) is pure crack resistance parameter, the energy including the new surfaces when the crack propagation is created in the inner fracture process zone (IFPZ). The second term is the non-essential (or plastic) work, (*W_p_*) corresponding to the outer “plastic” deformation zone (OPDZ). *W_e_* and *W_p_* are surface energy and plastic deformation energy, respectively. The value of *W_e_* is proportional to the ligament area (*tl*), and the value of *W_p_* is proportional to the volume of yielded area (*tl*^2^), Equation (1) can be expressed as the following:

*W_f_* = *w_e_ lt* + w*_p_βl*^2^*t* (2)

*w_f_* = *W_f_* ⁄ *lt* = *w_e_* + *βw_p_l* (3)

where *l* denotes the ligament length, *t* is the thickness of specimen, *β* is the shape factor whose value related to the geometry of the outer plastic zone, *w_e_* is called the specific essential work of fracture, it is materials’ nature property for a given thickness and independent of the sample dimension, and *w_p_* are specific non-essential work of fracture or specific plastic work. The data of specific essential work of fracture *w_f_* is determined on the ligament lengths (*l*) and appropriate dimensions of composites. The value of *w_f_* is plotted against *l*, that is to say, there must be a regression line, the intercept of regression line is *w_e_*, and the slope of the regression line is *βw_p_*.

## 3. Materials and Methods

### 3.1. Materials and Preparation

The BRF, BPF and WM were donated by the Chitianhua Paper Company Ltd. (Chishui, China). High-density polyethylene (HDPE) was purchased from Zhang Mu Tou Plastic Company Ltd. (Guangzhou, China). Its density is 0.945 g/cm^3^ (DGDK-3364), and melting mass flow rate is 0.75 g/10 min (190 °C, 2.16 kg). Maleic anhydride grafted polyethylene (MAPE), used as interfacial compatibilizer, was utilized to improve the compatibility between the polymer matrix and wood fiber, which was purchased from Zhang Mu Tou Company Ltd. (Guangzhou, China). The lubricant PE-Wax was used to improve lubricity during the processing of the BPCs, which is supplied by Yi-li Chemical Reagent Company (Beijing, China).

### 3.2. BPCs Manufacturing

The composites were prepared by a 45 mm conical twin-screw extruder (SJZ45/90-YF110; Kunshan, China). The volume fractions of the WM and BPF for the BPCs are illustrated in Table 1. In the first step, the raw materials were mixed according to the formulations listed in Table 1. The mixture were performed in a double-screw extruder (SJZ45/90-YF110), subjected to melt-blending, The temperatures of zones 1–4 were set at 160, 165, 175 and 175 °C, respectively. The temperature of the die was controlled at 180 °C, the extruded strand was passed through the die, which were air-cooled and then granulated with a crushing machine (ZJ300; Jiangyin, China). In the second step, the cooled granules were placed in the extruder (SJZ45/90-YF110) to produce the BPCs samples. 

### 3.3. BPF/BRF/WM Characterization

The length, diameter and length–diameter ratio of BPF (200 fibers) were measured with an optical microscope (Leica Microsystems, Wetzlar, Germany). Tensile strength of an individual BPF was measured using a specialized microtester (Instron Microtester 5848, Boston, MA, USA) according to the reference [27], which were designed for the short plant fibers. The tensile test with the load cell was 5 N, a gauge length of 10 mm was implemented on the Instron Microtester machine. The speed of crosshead was set at 0.048 mm/min. Tensile testing was carried out at 23 °C and 30% relative humidity. The collected data were calculated as the tensile properties of BPF. The micro morphology and diameters of BRF were observed with an optical microscope (Leica Microsystems). A Mastersizer 2000 (Malvern Instruments Ltd., Malvern, UK) laser particle size granularity analyzer was used to measure the particle size of WM.

### 3.4. Mechanical Testing and Characterization of the BPCs

All specimens were conditioned in a certain environment (23 °C, 30% RH) for 88 h according to ASTM D618-08.

Flexural property: Flexural strength and modulus for BPCs (160 mm× 14 mm × 8 mm) were conducted with a model 5582 Instron testing machine according to ASTM D790-10 (span = 200 mm). The values of the measured flexural properties were obtained from the average of six replicate specimens in each group.

Tensile property: The tensile samples of BPCs were cut into 100 mm × 10 mm × 3.5 mm pieces with dog-bone shaped and used for the tensile test, which was performed at a constant loading speed of 5 mm min^-1^ until failure under tension according to ASTM D638-10. The data recorded were used for calculating tensile strength and tensile modulus. The values of the measured tensile properties were obtained from the average of six replicate specimens in each group.

Impact strength: The notched impact strength of the BPCs were measured using a pendulum impact testing machine (XJJ-5, Chengde, China) according to ASTM D6110-10 at room temperature, which was provided by Chengde kecheng testing machine Co., Ltd. The values of the measured impact strength were obtained from the average of five replicate specimens in each group.

Impact EWF test: Double-edge notched impact samples were cut into dimensions of 150 mm × 12.7 mm × 8 mm from the BPCs used for this measurement. The notches of different depths were made by a milling machine and cut by using a razor blade. The exact ligament length (*l*) of impact samples were measured with vernier caliper. The impact EWF tests were measured with an impact test system at room temperature, which was conducted with a span length of 60 mm. The values of the measured impact EWF were obtained from the average of more than 10 replicate specimens in each group.

Microstructure: The microstructure of impact fracture surfaces of BPCs were observed using a JSM-6310F (Japan Co., Ltd., Tokyo, Japan) scanning electron microscope (SEM). The specimens were sputtered with gold coating to improve the surface conductivity before SEM observation, the SEM images were obtained at an acceleration voltage of 7.0 kV.

## 4. Results and Discussion

### 4.1. BPF Characterization

Table 2 shows the mean length, diameter and ratio of individual BPF. These properties affected the ultimate mechanical properties of BPCs.

The tensile property of BPF is presented in Table 3, the strength and the elastic modulus were 508.49 MPa and 6.73 GPa, respectively, while the elongation was 7.44%. These results were lower than that previously reported for bamboo fiber [28]. This may be due to the main constituents of bamboo cell wall being different, the contents of cellulose, hemicelluloses and lignin, which provide the specific mechanical properties and ultimately affect the properties of the BPF [27].

**Figure 2:**
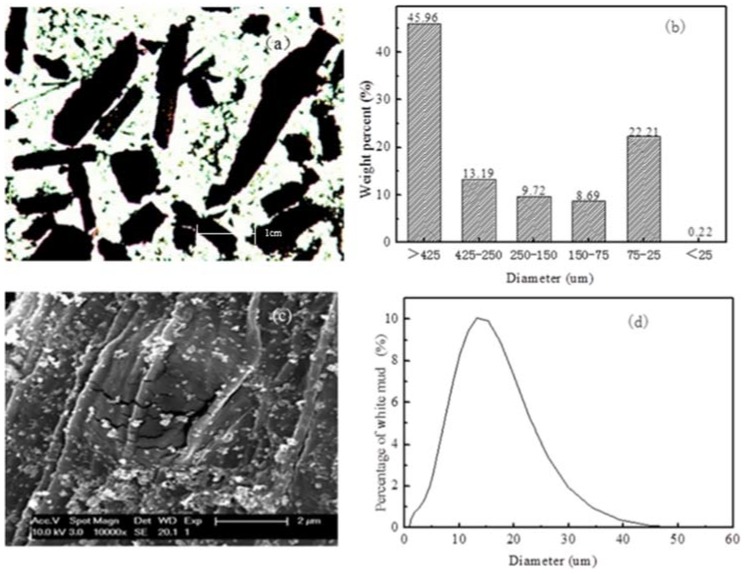
Analysis of bamboo residue: (**a**) optical micrograph of BRF at 50× magnification; (**b**) size of BRF; (**c**) SEM of WM at 10000 x magnification; (**d**) size of WM.

The analysis of BRF and WM are summarized in Figure 2. The morphology and diameter of BRF were observed with an FC300FX optical microscope (Leica Microsystems, Wetzlar, Germany) under 50× magnification (Figure 2a). It can be seen clearly from Figure 2a that the BRF particles were cylindrical, which has a lower aspect ratio compared to BPF. This may lead to poor dispersity of BRF in BPCs. Figure 2b shows that the diameter of BRF ranged from 75 to 425 μm. The scanning electron microscope (SEM) image of WM was shown in Figure 2c. Figure 2d shows the size distribution of WM. Maximum of the WM is 50 μm, while minimum WM particle size is approximately 700 nm, and the results showed that the diameter of WM was distributed in the range of 10 to 20 μm (Figure 2d). In other words, the WM was hybridized with micro/nano particles. 

Table 4 presents the summary of the chemical composition of WM. As it is shown, the primary chemical components of WM were calcium carbonate, and it is up to 87.97%.

### 4.2. Mechanical Properties of BPCs

**Figure 3:**
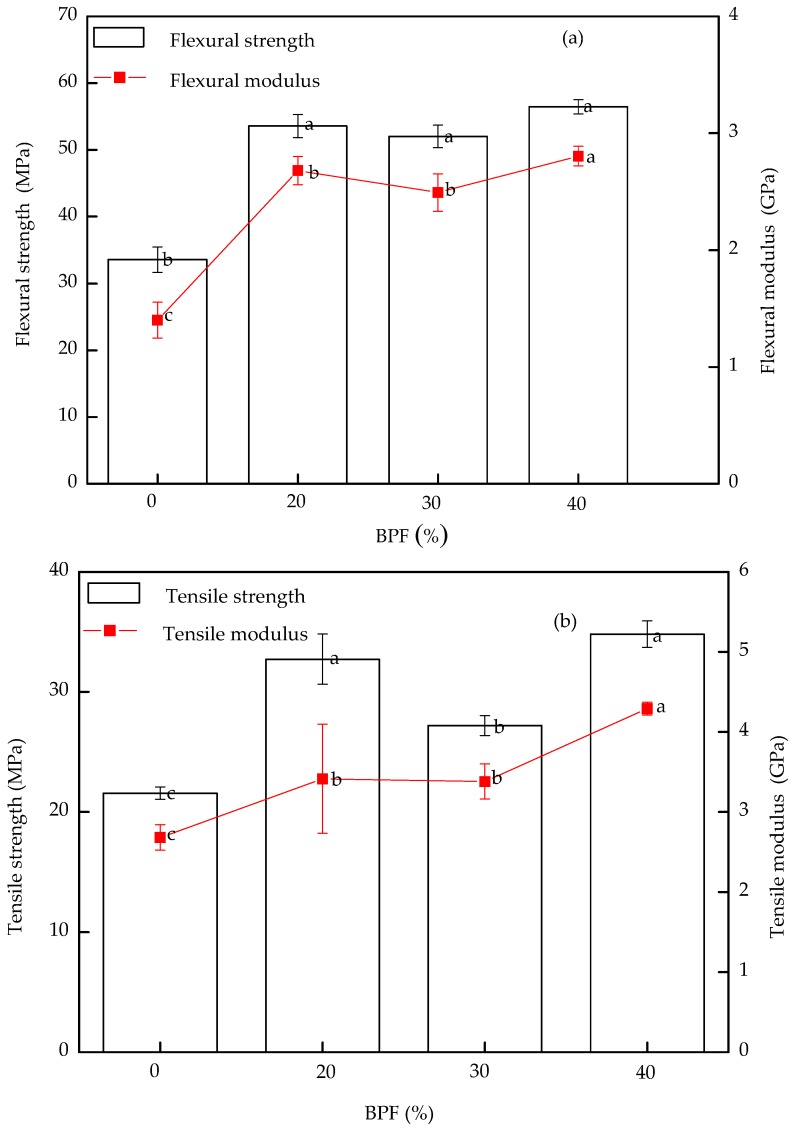

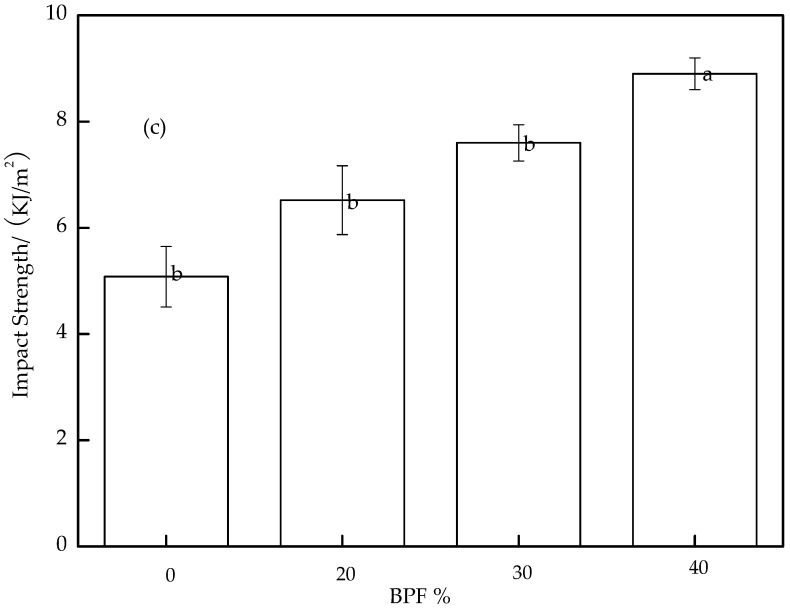
Effects of BPF on mechanical properties of BPCs: (**a**) effects of BPF on flexural property of BPCs; (**b**) effects of BPF on tensile property of BPCs; (**c**) effects of BPF on impact property of BPCs. Letters a, b, c denote significance test.

Flexural strength and modulus of BPCs are plotted in Figure 3a. The modulus of rupture (MOR) flexural strength values of BPF-reinforced BPCs exhibited an increasing trend with incorporation of BPF into BPCs. Flexural modulus of neat BRF-reinforced HDPE composites showed 1.46 GPa, and the flexural modulus of BPCs with 20% BPF was increased to 2.68 GPa. The addition of BPF tends to increase flexural modulus of BPCs which was due to high modulus/high aspect ratio of BPF acting as framework in the HDPE matrix [23]. Similar results were reported by otfher investigators [6,8]. For flexural strength, BPF-reinforced BPCs showed outstanding improvements with incorporation of BPF into BPC. When 20% of BPF was added, the flexural strength of BPCs was 1.60 times higher than that of without BPF-reinforced BPCs. Cross-bridged structure of BPF played an important role depending on the flexural strength of BPCs. The flexural strength and modulus of BPCs increased with increasing content of BPF, when BPF content was up to 40%, and it came to 56.47 MPa and 2.80 GPa, which were close to the 20% BPF loading in the BPCs. The results indicated the heterogeneous dispersion of the BPF and weak interface between BPF and HDPE. However, the flexural strength of BPCs is enhanced by the addition of BPF to some extent. In view of the cost, it was found that flexural property of the BPCs filled with the content of 20% BPF loading in composites is good.

The tensile properties of BPF-filled BPCs with different BPF concentration are shown in Figure 3b. It can be found that the tensile strength of BPCs was improved to some extent with adding 20% BPF to the BPCs. Comparing to the neat BRF-reinforced HDPE, it made an increase of 51.88%. After that, when 30% and 40% of BPF was added into the composites, the tensile strength of BPCs will increase to 26.17% and 61.53%, respectively, which indicated an improvement from tension transfer to the interface area between BPF and the matrix. These results indicated that loading BPF could improve the BRF–HDPE interfacial adhesion, the addition of BPF could enhance stress transfer efficiency from HDPE to BPF in BPCs, and after adding BPF, BPF and BRF, could be better crosslinked with HDPE so as to further improve tensile strengths of the BPCs. Similar to the flexural modules, tensile modulus of the samples surged with adding BPF from 0 to 40% (Figure 3b). When the additive amount of the BPF was 40%, the tensile modulus of the BPCs was 60.07% higher than the samples without BPF. These results indicated that the BPF was more rigid than the polymer in the BPCs. On the other hand, the motion and deformation capacity of the polymer matrix was restricted in the elastic zone after the addition of BPF. Thus, its elasticity modulus was higher than the composites without BPF.

Figure 3c shows the results of the effects of BPF on impact strength of BPCs. The addition of BPF into BRF-reinforced HDPE composites had positive effects on the impact strength of BPCs, which might be because BPF was well bonded to the HDPE matrix. Another reason was the large length–diameter ratio of BPF [23], acting as the HDPE framework, at the same time the BPF has less impact on the processability of HDPE matrix, which can absorb more impact energy. Interestingly, the notched impact strength of BPCs increased with increasing BPF content. When BPF content increases from 20% to 40%, the impact resistance of the BPCs improves by 28.35%, 49.61% and 75.19%, respectively. The results of impact strength indicated that the ductility and flexibility of BPCs increased after the BPF was added.

**Figure 4:**
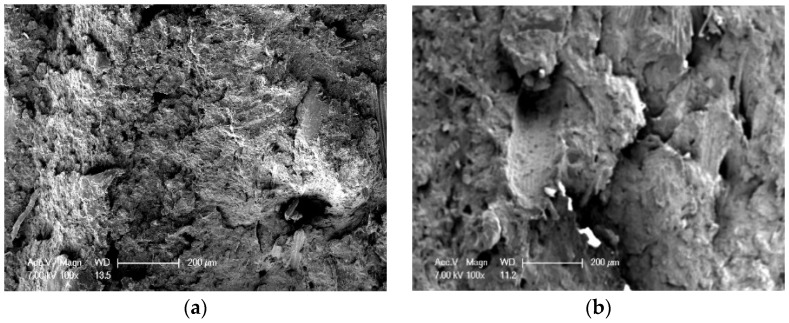

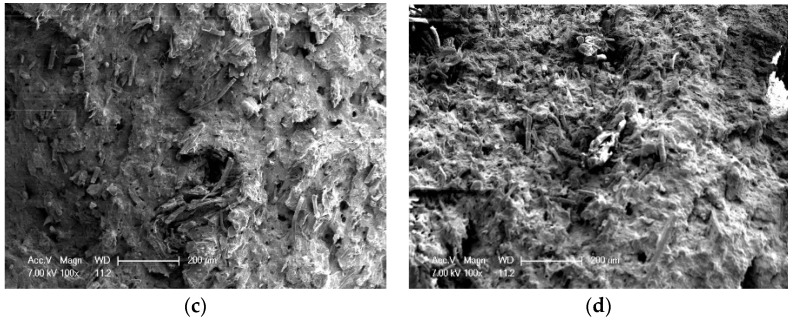
Microstructure of fracture surfaces of BPCs specimen: (**a**) containing 0% of BPF; (**b**) containing 20% of BPF; (**c**) containing 30% of BPF; (**d**) containing 40% of BPF.

Figure 4 shows SEM micrographs of the BPCs specimens, the fracture surface of the BPCs without addition of BPF; as shown in Figure 4a, it is indicated that the fracture is a typical brittle fracture. A few short fibers are observed in Figure 4b–d, it is clearly seen that there are some cavities on the surface of BPCs because of the absence of BPF. In addition, it was found that the fiber pull-out phenomenon occurred during the crack propagation for BPF-filled BPCs. This observation means that the fracture of BPF-reinforced BPCs is the fiber pull-out. Compared with control BPCs, it is found that the overall path length of initiated crack for BPF-filled BPCs is being enlarged because of this reason, resulting in a lot of energy needed to consume break the specimens [19]. The three main ways that composites absorbed energy are fiber pull-out, fiber breakage and matrix breakage. It is concluded that fiber pull-outs are the dominant toughening mechanisms for the BPF-reinforced BPCs in this study.

The flexural properties of WM-filled BPCs vary significantly with WM content. It is found that the flexural modulus of the BPCs increased with the growth of WM content in this study. This was due to the fact that the WM is rigid material compared to HDPE, according to the rule of mixtures. Such results occurred which restricted the motion of HDPE chains; as mentioned above, the flexural modulus of WM-filled BPCs was improved. This behavior of flexural properties was also observed in other experiments [29]. Generally, all the BPCs showed a flexural strength and modulus higher than the without WM-filled BPCs. It was found that BPCs with 14 wt % WM exhibited significantly higher flexural strength and modulus than those of BPCs without WM, which increased by 33.87% and 160.29%, respectively. This increase was expected due to the fact that the diameter of WM was much smaller than that of BRF, the tiny gaps between the BRF and HDPE would be filled by the micro/nano WM particles effectively, so that the internal structure of BPCs become compacted and WM particles distributed homogeneously in the BPCs. As a result, the flexural strength of the WM-filled BPCs increased instead.

**Figure 5:**
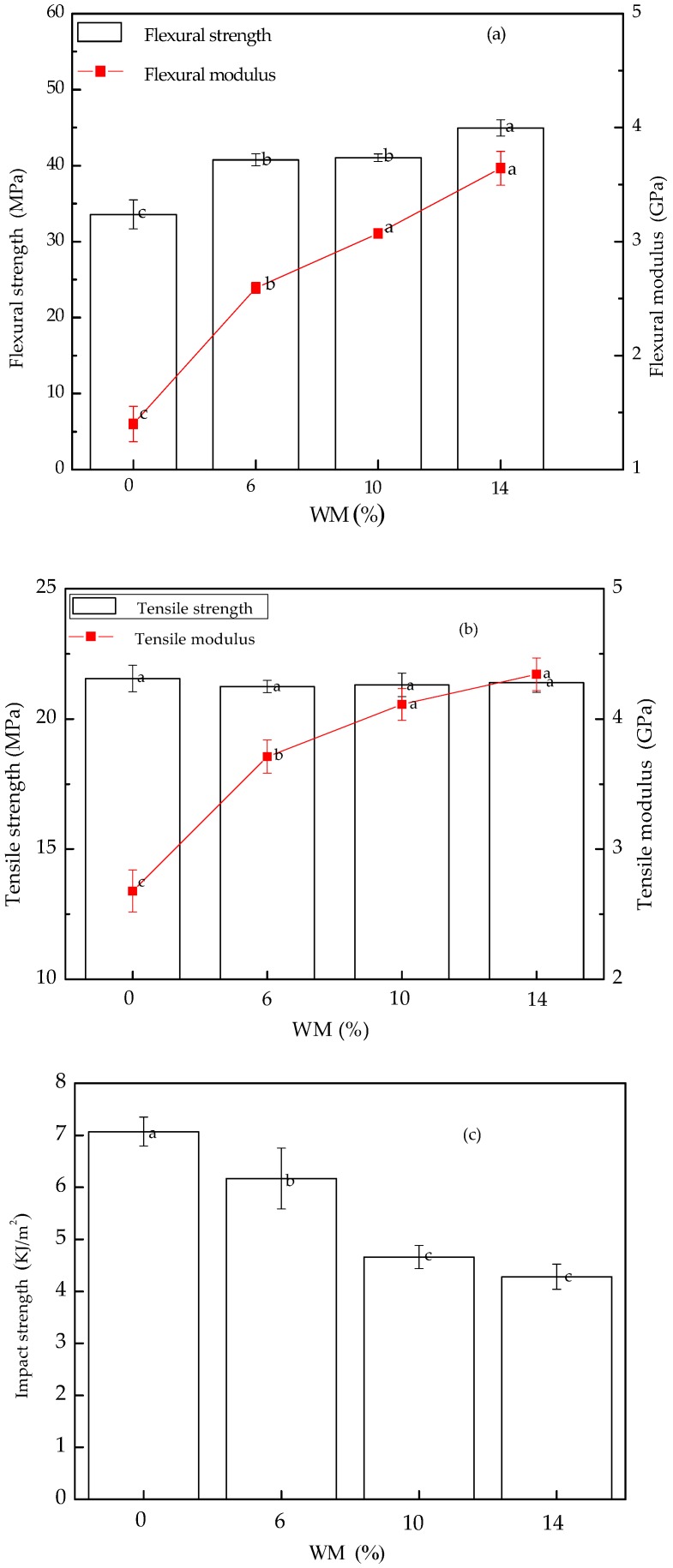
Effects of WM on mechanical properties of BPCs: (**a**) effects of WM on flexural property of BPCs; (**b**) effects of WM on tensile property of BPCs; (**c**) effects of WM on impact property of BPCs. Letters a, b, c denote significance test.

Figure 5b shows the tensile properties of WM-filled BPCs with various WM contents. The tensile modulus of BPCs increased with the addition of WM. This is due to the fact that the WM is inorganic rigid particles, which limited the motion of the polymer chains, and also decreased its plastic deformation capacity. The tensile modulus and strength of BPCs were 4.34 GPa and 21.39 MPa for 14 wt % WM employed. In other words, the tensile modulus of BPCs is enhanced 61.94% when the 14 wt % WM is in the BPCs. These results were already reported by previous researchers [23,29]. However, the test showed that the tensile strength of WM-filled BPCs was not changed with increasing the WM. There are numerous factors that affect tensile strength, such as nature of the reinforcement particles, particles size, interfacial adhesion, and also the distribution of particles in the BPCs [29]. 

**Figure 6:**
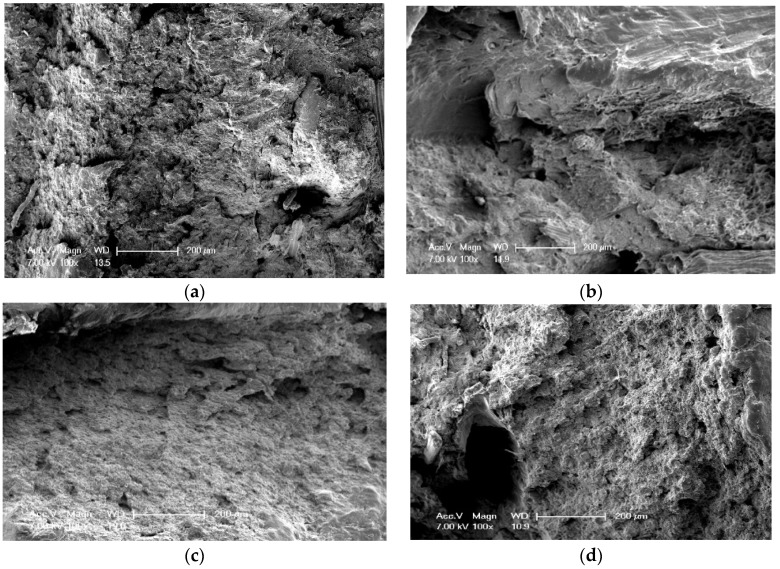
Microstructure of fracture surfaces of BPCs specimen: (**a**) containing 0% of BPF; (**b**) containing 6% of WM; (**c**) containing 10% of WM; (**d**) containing 14% of WM.

The impact property for the BPCs with different WM content is presented in Figure 5c. As WM particles were unevenly scattered in BPCs, they were unable to effectively terminate fracture initiation and fracture propagation to absorb impact energy when the BPCs were suffering hit from impact force [29,30]. The impact strength of WM-filled BPCs decreased significantly, due to the fact that the micro/nano WM filling effect was poor or hard to fill in tiny gaps of BRF and HDPE, yet they are profoundly incompatible and there were more local stress concentrations generated in the BPCs, so that cracks tend to grow and spread. This can be seen from SEM images of BPCs (Figure 6); it was found that no visual effectively filling was evident in the BPCs [31]. Besides, the compatibility between the WM and HDPE becomes poor with the increasing amount of WM, which led to unstable interfacial adhesions [32]. Consequently, the impact strength of BPCs decreased.

Figure 6 shows the fracture surfaces of BPCs specimens with different WM contents. A typical brittle fracture surface of BPCs can be found in Figure 6b, while containing 6 wt % WM particles in the BPCs, the presence of cavities which cracks tend to grow and spread when the particles were removed. According to the above impact strength results, it is reasonable to infer a conclusion that the fracture surface of BPCs has little or no contribution to the impact toughness of the specimen. Increasing the content of WM to 14 wt %, the feature of fracture surface is relatively flat. It also indicated that BPCs fractures is the brittle mode (Figure 6c,d). From the above observations, it results in stress concentration in the defects and matrix toughness decreases due to the addition of WM, which explains the brittle behavior of BPCs. A rough and irregular fractured surface is found and this means that the fractured interfacial area is weak between the WM and matrix polymer, which can be attributed to the impact fracture initiated and propagated trend.

### 4.3. EWF Analysis of Impact Test Results

**Figure 7:**
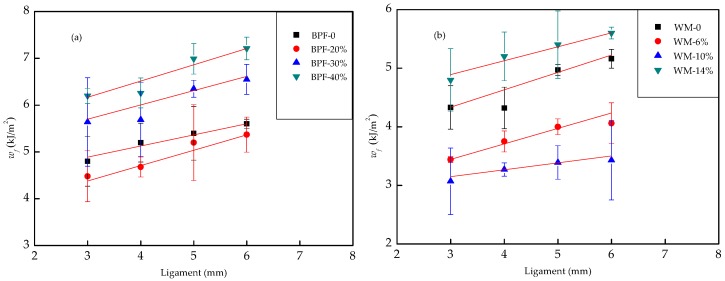
*w_f_* versus *l* (**a**) BPF-filled BPCs; (**b**) WM-filled BPCs.

Figure 7a,b shows the plots of impact fracture energy (*w_f_*) versus ligament length (*l*) for BPF-filled BPCs and WM-filled BPCs, respectively. It can be seen that the linear correlativity between the data of *w_f_* and *l* is obtained for these samples with a high determination coefficient. This indicated that the EWF method was relatively suitable for the BPCs. Following Equation (3), the value of *w_e_* was obtained from the interception, and representing the value of *βw_p_* from the slope of the straight lines. The results of impact EWF tests and the values of determination coefficient for all specimens investigated are presented in Table 5. According to the EWF theory equation, the total fracture energy of BPCs including two sections, one is dissipated in the inner region near the fracture surface completely, and another is dissipated in the outer plastic zone.

From Table 5, it was found that the specific essential fracture work (*w_e_*) values of BPF-filled BPCs notches impact tests were higher than WM-filled BPCs. The specific essential fracture work (*w_e_*) appears to increase with the addition of BPF. This result originates from the energy absorption effect of the BPF greatly enhancing during the crack propagation when BPCs are subjected to the impact force. In other words, the ability of stress transfer and crack propagation resistance depends mainly on the contribution from the addition of the BPF. This means that the length-diameter (L/D) ratio of BPF is largest and the rod-like shape leading to a larger interfacial area means that greater energy is required to initiate fracture. According to Figure 3c, the BPF loading has less effect on BPCs’ resistance. Meanwhile, WM-filled BPCs exhibit the lowest specific essential fracture work (*w_e_*) values, because of the uneven particle size and smaller interfacial area than that of BPF, leading to smaller energy dissipation in the interfacial area and also the stress concentration increases with increasing the WM loadings during the impact fracture process, which is confirmed from the SEM images.

The non-essential plastic work (*βw_p_*) of all BPCs samples is shown in Table 5. It was found that the fracture propagates are easy when the impact fracture is initiated. According to the experimental results, it was found that WM-filled BPCs present poor impact resistance during the fracture process, meaning that amount of WM leading to the non-uniform distribution in the BPCs. The non-essential plastic work (*βw_p_*) of BPF-filled BPCs did not show significant differences for the specimens; this indicated that the plastic deformation mechanism of composites is relatively consistent. This result is consistent with previous studies in an impact strength analysis study [33].

However, the *w_e_* and *βw_p_* values of the BPCs appear to decrease considerably by adding WM in comparison to the BPF-filled BPCs. This can be caused by the poor dispersibility of WM in the composites and weakens the interfacial interactions between the BRF and HDPE matrix. On the basis of EWF testing and the previous studies, it was found that the stiffness and toughness of the BPCs can be balanced by properly controlled raw materials contents [34]. As mentioned above, the presence of WM in the composites can improve the flexural property and tensile property of BPCs, but WM could decrease the impact strength of BPCs.

According to EWF analysis, there are two scenarios comparing BPF- and WM-filled BPCs in terms of the specific essential fracture work and the non-essential plastic work. One is both the specific essential fracture work and the non-essential plastic work values of BPCs (I) (for example BPF filled BPCs specimens) are higher than BPCs (II) (for example WM filled BPCs specimens), therefore BPCs (I) is recommended. Another is the specific essential fracture work values of BPCs (I) (for example BRF-HDPE specimens) are higher than BPCs (II) (for example 6% WM filled BPCs specimens), but the non-essential plastic work values of BPCs (II) are higher than BPCs (I). This means that BPCs (I) are hard to fracture initiation and easy to fracture propagation, but BPCs (II) are easy to fracture initiation and hard to fracture propagation. It proves that structure design or composites manufacturers should understand the practical connotations of impact behavior considering end-user applications, and also selecting the reasonable materials to be utilized.

## 5. Conclusions

The BPF and the WM are studied to evaluate and characterize the mechanical properties of BPCs. At the same time, the impact toughness of the BPC was analyzed based on EWF theory. Based on the results obtained, the conclusions can be drawn as following.

The filler BPF presents the best reinforcement to the BPCs. The flexural properties and tensile properties are increased at different extents for BPF used. The impact strength also sharply increased, the stiffness and strength of BPCs are significantly enhanced as the increased addition of WM, while the toughness of the BPCs is weakened. This is due to the fact that BPF has excellent strength and elastic modulus and the larger L/D ratio than WM particles, which means that BPF generates larger interfacial area. As a result, much of the energy dissipation occurs in the BPF-filled BPCs. Comparing to BPF-filled BPCs, WM particle is easy to crack propagation which contributes less energy dissipation. The scanning electron microscope (SEM) images of the BPCs were attributed to explain the different fracture of BPF- and WM-filled BPCs.

EWF analysis is feasible to analyze the impact strength of BPCs. The results showed that the specific essential work of fracture (*w_e_*) of BPCs was significantly increased with the addition of BPF, but decreased with the increase of WM; the contents of BPF had little effect on the specific non-essential work of fracture *βw_p_*. This result indicates that the fracture initiation and fracture propagation of BPCs are different. The results are useful for structure design or composites manufacturers to consider end-user applications, and also for selecting the reasonable materials to be utilized.

## Figures and Tables

**Table 1:** Formulation of bamboo plastic composites (BPCs).

Sample	BPF (%)	BRF (%)	WM (%)	HDPE (%)	MAPE (%)	PE-Wax (%)
1	20	30	0	45	4	1
2	30	20	0	45	4	1
3	40	10	0	45	4	1
4	0	50	0	45	4	1
5	50	0	6	39	4	1
6	50	0	10	35	4	1
7	50	0	14	31	4	1

Note: BPF: bamboo pulp fiber; BRF: bamboo residue fiber; WM: white mud; HDPE: High-density polyethylene; MAPE: maleic anhydride graft polyethylene; PE-wax: polyethylene wax.

**Table 2:** Initial length of bamboo fibers.

Sample	Length (μm)	Diameter (μm)	L/D Ratio
BPF	1146.61 (628.12)	17.49 (7.86)	63.10 (10.77)

Note: Standard deviations are in the parentheses; L/D: ratio of length to diameter.

**Table 3:** The tensile property of BPF.

Fiber	Cross-Sectional Area (μm^2^)	Fracturing Load (mN)	Tensile Strength (MPa)	Modulus (GPa)	Elongation at Break (%)
BPF	183.51 (17.68)	84.96 (19.54)	508.49 (162.27)	6.73 (5.11)	7.44 (0.77)

Note: Standard deviations are in the parentheses.

**Table 4:** Chemical constituents of white mud (%).

CaCO_3_	SiO_2_	Acid-Insoluble	CaO	NaOH	Fe_2_O_3_	Other
87.97	2.82	1.76	1.07	0.7	0.3	3.4

**Table 5:** Fracture parameters for the BPCs specimens.

Specimen	*w*_e_ (kJ/m^2^)	$\beta w_{p}\;(\mathrm{MJ}/m3)$	Impact Strength (kJ/m^2^)	*R* ^2^
BRF-HDPE	4.08	0.26	7.07	0.9485
BPF-20%	3.50	0.32	6.80	0.9360
BPF-30%	4.53	0.34	7.97	0.8521
BPF-40%	4.97	0.38	8.65	0.8540
WM-6%	3.28	0.31	6.17	0.8072
WM-10%	2.86	0.21	4.67	0.8966
WM-14%	2.75	0.12	4.28	0.8776
